# COVID-19 in Guangdong: Immediate Perceptions and Psychological Impact on 304,167 College Students

**DOI:** 10.3389/fpsyg.2020.02024

**Published:** 2020-08-12

**Authors:** Xueguo Li, Sihui Lv, Lili Liu, Rongning Chen, Jianbin Chen, Shunwei Liang, Siyao Tang, Jingbo Zhao

**Affiliations:** ^1^Mental Health Center, School of Public Health, Southern Medical University, Guangzhou, China; ^2^School of Management, Jinan University, Guangzhou, China; ^3^Department of Psychology, School of Public Health, Southern Medical University, Guangzhou, China

**Keywords:** COVID-19, stress, PTSS, college students, China

## Abstract

The outbreak of COVID-19 has brought unprecedented psychological pressure to people across China and more widely across the entire globe. The aim of this study was to assess the immediate perceptions of COVID-19 among college students in Guangdong Province, China, and to assess the psychological impact of the outbreak. We conducted a cross-sectional survey of college students via online questionnaires between February 13th and February 22nd, 2020. A total of 304,167 students completed the Impact of Event Scale 6 (IES-6) and other items. The results showed that 155,077 (50.9%) of the students reported stress symptoms, 1,565 (0.5%) reported poor mental health, and 9,752 (3.2%) reported poor sleep quality. Analysis indicated that the students’ perceptions of COVID-19 were correlated with psychological stress, self-perceived mental health and sleep quality. Moreover, the analysis revealed that the relationship between types of perception and levels of stress symptoms varied according to the students’ demographic characteristics. These findings allow us to better understand psychological stress among students and the factors influencing stress during the COVID-19 outbreak. Understanding these factors will help us to design intervention programs with the aim of alleviating stress among students and reducing the potential for developing psychological disorders.

## Introduction

A novel coronavirus was detected in Wuhan, Hubei Province, Central China, in December 2019, and on the 30th of January 2020 the World Health Organization declared the outbreak to be a Public Health Emergency of International Concern.^1^ The COVID-19 virus has now spread to countries across the globe, the world is facing an unprecedented challenge with communities and economies everywhere affected by the growing pandemic. By 8 March 2020, a total number of 1,352 confirmed cases were confirmed in Guangdong Province, making it the second worst-affected province in China after Hubei.^2^ All age groups can catch COVID-19, older people and people with underlying medical problems like cardiovascular disease, diabetes and chronic respiratory disease are more likely to develop serious illness. At this time, there are no specific vaccines or treatments for COVID-19. The temporary case fatality rate by WHO is about 2%, but some researchers estimate that the rate is between 0.3% and 0.6% (Nishiura et al., 2020). COVID-19 poses a constant threat to human health with its high level of transmission, severe consequences of infection, and the uncertain duration of the epidemic. Following the outbreak of COVID-19, the Chinese government implemented a series of strong epidemic prevention measures, and all parts of the country had launched a Level I response to public health emergencies by January 29, 2020.

Empirical research has found that public health emergencies and disasters often cause great psychological stimuli to those affected. Studies on health emergencies and catastrophes such as the Wenchuan earthquake, Ebola outbreak and SARS found that such events can cause mental health problems (Chit et al., 2009; Shultz et al., 2015). Once the stimulus exceeds an individual’s general psychological response level it can bring serious consequences, including cognitive changes, emotional changes, physical reactions and behavioral changes (Lu et al., 2006; Lee et al., 2007; Huang and Zhao, 2020). However, the characteristics of an epidemic outbreak and a general catastrophic event are very different. The location and scope of a general catastrophic event are determined, and the duration is often transient. However, the occurrence of an epidemic is uncertain in time and space. This uncertainty is more likely to lead to an imbalance in the mindset of the general public, a loss in the sense of security and control, a state of stress, tension, anxiety, confusion, and even hopelessness (Rubin et al., 2010; Wang et al., 2020b). Faced with an emergency, people’s initial psychological tension is normal. It enables local authorities to raise awareness of self-prevention, to strengthen prevention capabilities and to implement preventive measures (Leung et al., 2003). However, increasing numbers of confirmed and suspected cases and disease trends in the news can increase people’s stress, and some exhibit stress reactions such as fear and nervousness. Due to the prevalence of ‘We Media,’ the variety of media reports on COVID-19, and restrictions on personal knowledge, individuals may pay excessive attention to the potential risk factors and exaggerate their degree, keeping themselves in a state of continuous stress (Rubin and Wessely, 2020). Long-term excessive stress will cause autonomic and endocrine dysfunction, which will further affect and weaken the immune functions of the human body, leading to a decline in the body’s ability to resist disease (Miller and Cohen, 2001). Often this psychological stress response is composed of negative cognitive emotional states (Wang et al., 2020b).

The Chinese government instituted mitigation policies to contain the spread of the epidemic. For example, confirmed cases were quarantined, suspicious cases were monitored and restricted by house arrest, universities remained closed, and people were asked to stay at home as much as possible (Hellewell et al., 2020). These actions are a very effective way to interrupt the transmission of the virus. However, they may lead to more serious mental health problems (Brooks et al., 2020). The prevention and control of the epidemic situation requires people’s activities to be restricted or strictly restricted for a long period of time, which affects their normal life, work and study. Moreover, these prevention measures may bring about torment, irritability, or an emotional state of boredom, helplessness or hopelessness. To address such mental health issues in China, the National Health Commission of China has released guidelines for local authorities to promote psychological crisis intervention measures during the COVID-19 outbreak for patients, medical staff, and people under medical observation.^3^ However, the outbreak of COVID-19 has also caused mental health problems among the general public in China (Bao et al., 2020). A rapid assessment was conducted via an online survey of 4,872 Chinese citizens, and the findings showed a high prevalence of mental health problems among the general population, especially depression and anxiety (Gao J. et al., 2020). It was also seen that psychological and behavioral responses to COVID-19 had been dramatic during the rising phase of the outbreak (Qiu et al., 2020).

Many studies have highlighted mental health issues in young adults, especially during their years at university (Blanco et al., 2008; Milojevich and Lukowski, 2016; Saleh et al., 2017). At present, there are 33.66 million college students in China, of whom 8.83 million are inter-provincial students. During the outbreak, colleges and universities announced the postponement of school openings, which is helpful for epidemic control (Wang et al., 2020a). Stressors such as fear of infection, prolonged duration, study maladjustment and family financial loss can cause even more mental health problems for college students. College students are a special group of people who accept new things easily, have multiple channels for information acquisition, and have high levels of social media activity. One the one hand, there are some studies showed that up-to-date and accurate health information was associated with lower stress levels (Holmes et al., 2020; Wang et al., 2020b). On the other hand, young people tend to obtain a large amount of information from social media which can easily trigger stress if too much time is spent on information about the corona epidemic (Qiu et al., 2020). Recent research has also found a higher prevalence of depressive symptoms among the young (Huang and Zhao, 2020). Faced with the spread of epidemic information from various sources, college students are more likely to develop disease information deviations, such as catastrophic cognition of the disease, high self-risk perception, an underestimation of their self-coping ability, and cognitive inconsistency, which may contribute to the psychological impact. However, it is unclear how college students perceive this epidemic and how their perception affects their mental health. An assessment of outbreak-associated perceptions of COVID-19 and the psychological impact on students is therefore an urgent need.

The current study conducted a survey of students in 85 colleges in Guangdong Province on February 13–22, 2020, in order to understand the perceptions of COVID-19 among students and to analyze the relations between perceptions and their recent mental health status. We proposed the following hypotheses. Firstly, the more negative the perception, the greater the psychological impact of the epidemic. Secondly, when reality or behavior is inconsistent with perception, the psychological impact is large; but when real events are consistent with perception the psychological impact is small. The aim is to explore the current status of college students’ perceptions of COVID-19 and the impact of related factors on their mental health, with the goal of providing information for the formulation of group psychological precautions and related work in universities.

## Materials and Methods

### Study Design and Participants

A cross-sectional survey covering students from 85 colleges in Guangdong Province was conducted between February 13th and February 22nd, 2020, 2 weeks after the Ministry of Education issued a notice on January 27th 2020 on the postponement of the spring semester. An online questionnaire consisted of the participant’s informed consent, baseline sociodemographic information, perceptions of the threat of COVID-19, psychological protective measures and rating scales, including the Impact of Event Scale 6 (IES-6).

The online questionnaire was distributed to all directors of the mental health centers of 85 colleges via WeChat. The directors in each college were responsible for the distribution and collection of the questionnaires. Only one response per person was permitted. The senior investigators performed quality control by checking the collected questionnaires daily. The study was approved by the institutional ethics board of Southern Medical University.

### Measures

#### Demographic Information

Participants provided demographic information on their age, gender, grade, nationality, family location, history of physical illness, psychiatric history, counseling history, information about their perceived mental health, and whether there had been diagnosed or suspected cases of COVID-19 infection in their friends, relatives and families.

#### Perceptions

Two items assessed perceived threats of COVID-19. Participants were asked ‘Do you think that COVID-19 infection can be prevented?’ and ‘Do you think that COVID-19 infection can be treated?’ Another item asked, ‘Since the COVID-19 epidemic, have you taken the precautions you have learned?’ to assess whether they had taken action.

#### Level of Posttraumatic Stress Symptoms IES-6

The Impact of Event Scale 6 is a useful screening instrument for research in epidemiological studies or in clinical practice, simplified by Thoresen on the basis of the Impact of Events Scale Revised (IES-R) and highly correlated to IES-R (Thoresen et al., 2010). IES-6 is a 6-item self-report measure of psychological response to trauma, with each item rated on a scale from 0 to 4. Its three subscales (Intrusion, Avoidance, and Hyperarousal) are closely affiliated with PTSD symptoms. It can be anchored to any specific event, such as the COVID-19 epidemic. The average score *S* of IES-6 is categorized as follows: *S* < 1.09 = normal; 1.09 ≤ *S* < 1.5 = showing stress symptoms; *S* ≥ 1.5 = may be diagnosed with PTSD (Jalloh et al., 2018). In this study, for the purpose of detecting the presence of posttraumatic stress symptoms (PTSS) rather than diagnostic PTSD, we used an average score of 1.09 or greater as a cutoff for significant stress. The Cronbach’s alpha for the IES-6 in the current sample was 0.782, and the composite reliability was 0.801.

#### Levels of Other Psychological Impact

Three items were used to assess the physical health, mental health, and sleep quality of participants in isolation at home during the outbreak. Participants were asked, ‘How is your physical health?’, ‘How is your mental health?’ and ‘Overall, how would you rate your sleep quality during the novel coronavirus epidemic?’ A five-point scale rated responses from 1 (very good) to 5 (very poor).

### Statistical Analysis

Data were analyzed using SPSS Version 22.0. Descriptive statistics were conducted to characterize the sample’s demographic profile and level of psychological stress. A reliability test was used to check the internal consistency of IES-6. Normality of quantitative data was checked using the One-Sample Kolmogorov–Smirnov Test. The results showed that the levels of psychological impact were non-normal continuous variables. Differences among groups were tested by the Mann–Whitney *U* test or the Kruskal–Wallis *H* test for non-normal continuous variables, and by a chi-square test or Fisher’s Exact test for categorical variables whenever appropriate. Univariate test was used to test the interaction between demographic variables and perception types. Values of *p* < 0.05 (two-tailed tests) were considered statistically significant.

## Results

### Demographic Characteristics of Participants

Overall, 361,969 participants answered the questionnaire. Among these participants, 19,322 were ineligible because of living outside Guangdong Province and 38,480 questionnaires were invalid, leaving 304,167 respondents who completed the questionnaire successfully. The response rate was 84.0% (304,167/361,969). All 304,167 participants were from colleges in Guangdong Province and lived in Guangdong.

Of the 304,167 participants, 59.9% were female and the majority (84.4%) were 19–22 years old. A total of 155,077 respondents (50.9%) were identified as having psychological stress symptoms. However, the majority rated their physical health as “very good” (69.3%) or “good” (25.7%). The majority also rated their sleep quality as “very good” (37.7%) or “good” (37.3%). Table 1 presents the baseline characteristics for the total sample (*n* = 30,417) and for the sample divided into four possible perceptions of COVID-19: those students who thought that COVID-19 infection could be prevented and that the disease was treatable (the ‘preventable and curable’ group) (*n* = 290,148); those who thought that infection could be prevented but the disease was not treatable (the ‘preventable and incurable’ group) (*n* = 5,549); those who thought that infection was not preventable but the disease was treatable (the ‘unpreventable and curable’ group) (*n* = 6,979); and those who thought that infection was unpreventable and the disease was untreatable (the ‘unpreventable and incurable’ group) (*n* = 1,491).

**TABLE 1:** Demographic variables and perception groups.

Variable (*n* = 304,167)	All participants (%)	Preventable and curable group (%)	Preventable and incurable group (%)	Unpreventable and curable group (%)	Unpreventable and incurable group (%)	χ ^2^	df	*p*
**Gender**								
Male	122102 (40.1)	116289 (40.1)	2367 (42.7)	2600 (37.3)	846 (56.7)	210.247	3	<0.001
Female	182065 (59.9)	173859 (59.9)	3182 (57.3)	4379 (62.7)	645 (43.3)			
**Nationality**								
The Han nationality	301657 (99.2)	287773 (99.2)	5500 (99.1)	6910 (99.0)	1474 (98.9)	4.470	1	0.215
Minority nationality	2510 (0.8)	2375 (0.8)	49 (0.9)	69 (1.0)	17 (1.1)			
**Age group**								
≤18	26918 (8.8)	25742 (8.9)	463 (8.3)	579 (8.3)	134 (9.0)	62.359	9	<0.001
19–22	256817 (84.4)	245087 (84.4)	4624 (83.3)	5877 (84.2)	1229 (82.4)			
23–25	19767 (6.5)	18699 (6.4)	446 (8.0)	506 (7.3)	116 (7.8)			
≥26	665 (0.2)	620 (0.2)	16 (0.3)	17 (0.2)	12 (0.8)			
**Grade**								
Fresher	121380 (39.9)	116318 (40.1)	2000 (36.0)	2547 (36.5)	515 (34.5)	150.807	9	<0.001
Sophomore	96964 (31.9)	92513 (31.9)	1713 (30.9)	2238 (32.1)	500 (33.5)			
Junior	63265 (20.8)	60027 (20.7)	1327 (23.9)	1566 (22.4)	345 (23.1)			
Senior (4th and 5th)	22558 (7.4)	21290 (7.3)	509 (9.2)	628 (9.0)	131 (8.8)			
**Family location**								
Rural areas	123351 (40.6)	117868 (40.6)	2227 (40.1)	2703 (38.7)	553 (37.1)	32.340	6	<0.001
County town	92490 (30.4)	88288 (30.4)	1652 (29.8)	2087 (29.9)	463 (31.1)			
City	88326 (29.0)	83992 (28.9)	1670(301.)	2189 (31.4)	475 (31.9)			
**Family member (co-resident)**								
1–2 people	12643 (4.2)	11956 (4.1)	277 (5.0)	326 (4.7)	84 (5.6)	41.150	9	<0.001
3people	56534 (18.6)	53920 (18.6)	987 (17.8)	1331 (19.1)	296 (19.9)			
4 people	102447 (33.7)	97896 (33.7)	1764 (31.8)	2324 (33.3)	463 (31.1)			
5 people and above	132543 (43.6)	126376 (43.6)	2521 (45.4)	2998 (43.0)	648 (43.5)			
**Daily time spend on reading information about COVID-19**								
≤1 h	157258 (51.7)	149228 (51.4)	3127 (56.4)	3993 (57.2)	910 (61.0)	381.506	6	<0.001
1–3 h	115231 (37.9)	110944 (38.2)	1784 (32.1)	2150 (30.8)	353 (23.7)			
≥3 h	31678 (10.4)	29976 (10.3)	638 (11.5)	836 (12.0)	228 (15.3)			
**Severe physical illness**								
Yes	932 (0.3)	842 (0.3)	25 (0.5)	42 (0.6)	23 (1.5)	100.794	3	<0.001
No	303235 (99.7)	289306 (99.7)	5524 (99.5)	6937 (99.4)	1468 (98.5)			
**History of mental illness**								
Yes	2187 (0.7)	1989 (0.7)	71 (1.3)	92 (1.3)	35 (2.3)	119.474	3	<0.001
No	301980 (99.3)	288159 (99.3)	5478 (98.7)	6887 (98.7)	1456 (97.7)			
**History of counseling**								
Yes	11442 (3.8)	10677 (3.7)	315 (5.7)	352 (5.0)	98 (6.6)	125.808	3	<0.001
No	292725 (96.2)	279471 (96.3)	5234 (94.3)	6627 (95.0)	1393 (93.4)			
**Infection of friends**								
Someone is diagnosed	491 (0.2)	1661 (0.6)	73 (1.3)	81 (1.2)	57 (3.8)	1398.378	6	<0.001
Someone is suspected	1381 (0.5)							
No one is infected	198653 (65.3)	191240 (65.9)	3135 (56.5)	3703 (53.1)	575 (38.6)			
Not sure	103642 (34.1)	97247 (33.5)	2341 (42.2)	3195 (45.8)	859 (57.6)			
**Infection of relatives (non co-resident)**								
Someone is diagnosed	244 (0.1)	610 (0.2)	28 (0.5)	37 (0.5)	27 (1.8)	2080.461	6	<0.001
Someone is suspected	458 (0.2)							
No one is infected	276063 (90.8)	264649 (91.2)	4704 (84.8)	5721 (82.0)	999 (66.3)			
Not sure	27402 (9.0)	24889 (8.6)	817 (14.7)	1221 (17.5)	475 (31.9)			
**Infection of families (co-resident)**								
Someone is diagnosed	83 (0.0)	245 (0.1)	14 (0.3)	15 (0.2)	15 (1.0)	2756.788	6	<0.001
Someone is suspected	206 (0.1)							
No one is infected	294847 (96.9)	282097 (97.2)	5198 (93.7)	6487 (91.5)	1165 (78.1)			
Not sure	9031 (3.0)	7806 (2.7)	337 (6.1)	577 (8.3)	311 (20.9)			
**Taking precautions**								
Yes	294930 (97.0)	283122 (97.6)	5166 (93.1)	5729 (82.1)	913 (61.2)	12362.086	3	<0.001
No	9237 (3.0)	7026 (2.4)	383 (6.9)	1250 (17.9)	578 (38.8)			
**Self -perceived physical health**								
Very good	210938 (69.3)	202322 (69.7)	3438 (62.0)	4307 (61.7)	871 (58.4)	1705.798	12	<0.001
Good	78176 (25.7)	74254 (25.6)	1574 (28.4)	1987 (28.5)	361 (24.2)			
General	14440 (4.7)	13082 (4.5)	504 (9.1)	636 (9.1)	218 (14.6)			
Poor	511 (0.2)	414 (0.1)	26 (0.5)	42 (0.6)	29 (1.9)			
Very poor	102 (0.0)	76 (0.0)	7 (0.1)	7 (0.1)	12 (0.8)			
**Self-perceived mental health**								
Very good	203467 (66.9)	195662 (67.4)	3145 (56.7)	3901 (55.9)	759 (50.9)	3184.494	12	<0.001
Good	78363 (25.8)	74444 (25.7)	1591 (28.7)	1993 (28.6)	335 (22.5)			
General	20771 (6.8)	18760 (6.5)	726 (13.1)	968 (13.9)	317 (21.3)			
Poor	1263 (0.4)	1056 (0.4)	73 (1.3)	94 (1.3)	40 (2.7)			
Very poor	303 (0.1)	226 (0.1)	14 (0.3)	23 (0.3)	40 (2.7)			
**Sleeping quality**								
Very good	114550 (37.7)	110131 (38.0)	1732 (31.2)	2216 (31.8)	471 (31.6)	2492.957	12	<0.001
Good	113388 (37.3)	109035 (37.6)	1786 (32.2)	2238 (32.1)	329 (22.1)			
General	66477 (21.9)	62339 (21.5)	1642 (29.6)	2031 (29.1)	465 (31.2)			
Poor	7649 (2.5)	6897 (2.4)	271 (4.9)	361 (5.2)	120 (8.0)			
Very poor	2103 (0.7)	1746 (0.6)	118 (2.1)	133 (1.9)	106 (7.1)			
**IES level**								
Normal	149090 (49.0)	143078 (49.3)	2359 (42.5)	3052 (43.7)	601 (40.3)	377.585	6	<0.001
Stress symptoms	55499 (18.2)	53098 (18.3)	973 (17.5)	1203 (17.2)	225 (15.1)			
Diagnostic PTSD	99578 (32.7)	93972 (32.4)	2217 (40.0)	2724 (39.0)	665 (44.6)			

### Demographics and Perceptions of COVID-19

Participants who were 19–22 years old and participants who were freshers had significantly more chance of being in the ‘preventable and curable’ group (χ^2^ = 62.359, df = 9, *p* < 0.001; χ^2^ = 150.807, df = 9, *p* < 0.001). Participants who were 23–25 years old, those who were junior or senior students, those who lived in city, those who lived alone or lived with only one family member, those who had a severe physical illness, those who had a history of mental illness, and those who had a history of counseling had significantly less chance of being in the ‘preventable and curable’ group (χ^2^ = 62.359, df = 9, *p* < 0.001; χ^2^ = 150.807, df = 9, *p* < 0.001; χ^2^ = 32.340, df = 6, *p* < 0.001; χ^2^ = 41.150, df = 9, *p* < 0.001; χ^2^ = 100.794, df = 3, *p* < 0.001; χ^2^ = 119.474, df = 3, *p* < 0.001; χ^2^ = 125.808, df = 3, *p* < 0.001). Participants whose friends, relatives or families were not infected with COVID-19 had significantly more chance of being in the ‘preventable and curable’ group, followed by a significantly greater chance of being in the ‘preventable and incurable’ group (χ^2^ = 1398.378, df = 6, *p* < 0.001; χ^2^ = 2080.461, df = 6, *p* < 0.001; χ^2^ = 2756.788, df = 6, *p* < 0.001). The ‘preventable and curable’ group were significantly more likely to take precautions and had a significantly higher chance of having very good self-perceived physical health, mental health and sleep quality (χ^2^ = 12362.086, df = 3, *p* < 0.001; χ^2^ = 1705.798, df = 12, *p* < 0.001; χ^2^ = 3184.494, df = 12, *p* < 0.001; χ^2^ = 2492.957, df = 12, *p* < 0.001).

Participants who were male, those who were older than 26 years, those who were sophomores, those with a severe physical illness, those who had a history of mental illness, those who had a history of counseling, and those who spent 3 h or more on reading information about COVID-19 every day had significantly more chance of being in the ‘unpreventable and incurable’ group (χ^2^ = 210.247, df = 3, *p* < 0.001; χ^2^ = 62.359, df = 9, *p* < 0.001; χ^2^ = 150.807, df = 9, *p* < 0.001; χ^2^ = 100.794, df = 3, *p* < 0.001; χ^2^ = 119.474, df = 3, *p* < 0.001; χ^2^ = 125.808, df = 3, *p* < 0.001; χ^2^ = 381.506, df = 6, *p* < 0.001). Participants whose friends, relatives or families were diagnosed or suspected with COVID-19 had significantly more chance of being in the ‘unpreventable and incurable’ group. Those who did not know the infection status of their friends, relatives and families had significantly more chance of being in the ‘unpreventable and incurable’ group, followed by a significantly greater chance of being in the ‘unpreventable and curable’ group (χ^2^ = 1398.378, df = 6, *p* < 0.001; χ^2^ = 2080.461, df = 6, *p* < 0.001; χ^2^ = 2756.788, df = 6, *p* < 0.001). The ‘unpreventable and incurable’ group were significantly more likely to take no precautions and had a significantly higher chance of having moderate, poor or very poor self-perceived physical health, mental health and sleep quality (χ^2^ = 12362.086, df = 3, *p* < 0.001; χ^2^ = 1705.798, df = 12, *p* < 0.001; χ^2^ = 3184.494, df = 12, *p* < 0.001; χ^2^ = 2492.957, df = 12, *p* < 0.001). The number of students who may be diagnosed with PTSD in the ‘unpreventable and incurable’ group was much higher than those in other groups (χ^2^ = 377.585, df = 6, *p* < 0.001).

Participants with good self-perceived physical health and mental health had significantly more chance of being in the ‘preventable and incurable’ group or the ‘unpreventable and curable’ group (χ^2^ = 1705.798, df = 12, *p* < 0.001; χ^2^ = 3184.494, df = 12, *p* < 0.001). Female participants had significantly more chance of being in the ‘unpreventable and curable’ group (χ^2^ = 210.247, df = 3, *p* < 0.001).

### Associations Between Perceptions and Psychological Impact

Table 2 compares the results for psychological stress symptoms, self-perceived mental health and sleeping quality among the four groups. Levels of stress and intrusion were significantly highest in the ‘unpreventable and incurable’ group (χ^2^ = 413.532, *p* < 0.001; χ^2^ = 563.690, *p* < 0.001). Self-perceived mental health and sleeping quality were significantly poorer in the ‘unpreventable and incurable’ group (χ^2^ = 599.833, *p* < 0.001; χ^2^ = 887.284, *p* < 0.001). In sum, the comparison reveals that the ‘unpreventable and incurable’ group had experienced a greater psychological impact whereas the ‘preventable and curable’ group suffered less.

**TABLE 2:** Comparisons of psychological impact.

Variables	Preventable and curable group①	Preventable and incurable group②	Unpreventable and curable group③	Unpreventable and incurable group④	χ ^2^	*p*	
Item 1 of IES-6	1.47 ± 0.97	1.60 ± 1.08	1.58 ± 1.08	1.55 ± 1.21	128.198	<0.001	① < ②③④
Item 2 of IES-6	2.30 ± 1.07	2.36 ± 1.11	2.24 ± 1.13	2.06 ± 1.29	95.420	<0.001	④ < ③ < ① < ②
Item 3 of IES-6	1.38 ± 1.01	1.54 ± 1.10	1.50 ± 1.10	1.56 ± 1.21	179.374	<0.001	① < ②③④
Item 4 of IES-6	0.80 ± 0.94	1.00 ± 1.06	1.02 ± 1.07	1.21 ± 1.19	605.927	<0.001	① < ②③<④
Item 5 of IES-6	0.72 ± 0.94	0.85 ± 1.04	0.86 ± 1.05	1.08 ± 1.15	327.391	<0.001	① < ②③<④
Item 6 of IES-6	0.43 ± 0.75	0.62 ± 0.94	0.63 ± 0.92	0.90 ± 1.10	1021.087	<0.001	① < ②③<④
IES-6	7.09 ± 3.91	7.97 ± 4.51	7.82 ± 4.52	8.36 ± 5.27	413.532	<0.001	① < ②③<④
Avoidance	1.52 ± 1.63	1.84 ± 1.85	1.88 ± 1.86	2.28 ± 2.10	563.690	<0.001	① < ②③<④
Intrusion	2.84 ± 1.75	3.14 ± 1.95	3.08 ± 1.94	3.12 ± 2.16	198.165	<0.001	① < ②③④
Hyperarousal	2.73 ± 1.39	2.98 ± 1.58	2.87 ± 1.58	2.96 ± 1.87	168.956	<0.001	① < ③ < ②, ① < ④
Self -perceived mental health	1.40 ± 0.63	1.60 ± 0.78	1.62 ± 0.80	1.84 ± 1.02	599.833	<0.001	① < ②③<④
Sleep quality	1.90 ± 0.86	2.15 ± 0.99	2.13 ± 0.98	2.37 ± 1.21	887.284	<0.001	① < ②③<④

*Items of IES-6: 1. I thought about it when I didn’t mean to; 2. I felt watchful or on-guard; 3. Other things kept making me think about it; 4. I was aware that I still had a lot of feelings about it, but I didn’t deal with them; 5. I tried not to think about it; 6. I had trouble concentrating.*

### Associations Between Demographics, Perceptions, and Psychological Stress

**FIGURE 1:**
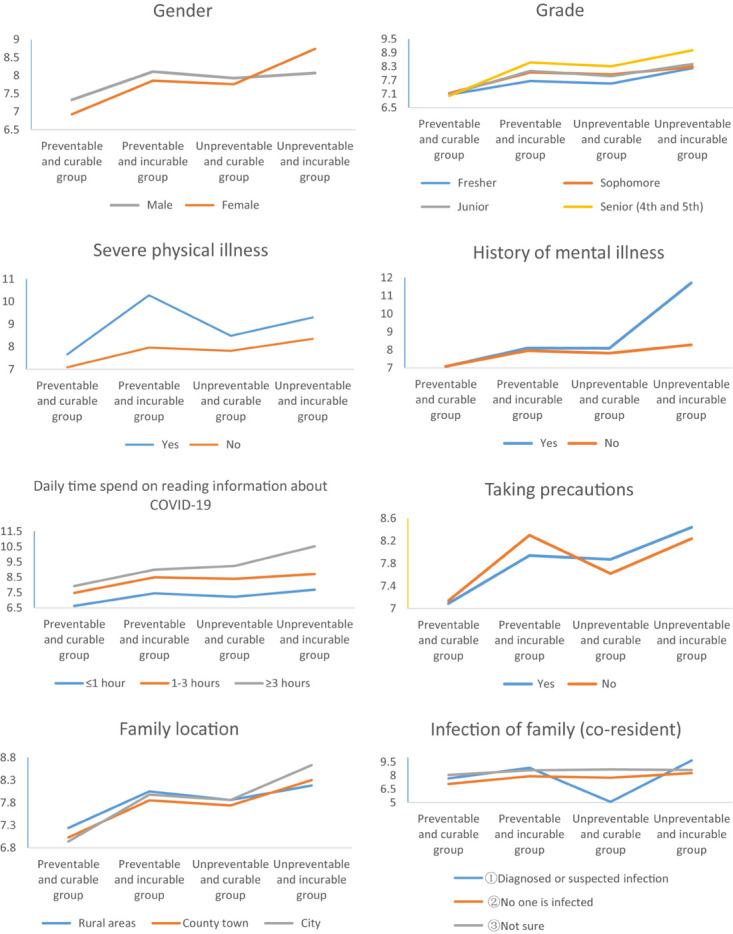
Associations between psychological stress symptoms, demographic data, and perceptions.

The authors explored further the associations between psychological stress, demographic data and perceptions of COVID-19 (Table 3). There were main effects of perceptions on all demographics, indicating that higher IES-6 scores for ‘unpreventable and incurable’ group than other groups when controlled gender, history of mental illness, history of physical illness, infection condition of people around, family location, family members and daily time to read information about COVID-19, and that lower IES-6 scores for the ‘preventable and curable’ group than other groups when controlled demographic variables listed in Table 3. Figure 1 shows these effects.

**TABLE 3:** Associations between psychological stress symptoms, demographic data, and perceptions of COVID-19.

**Variables**	**Preventable and curable group**	**Preventable and incurable group**	**Unpreventable and curable group**	**Unpreventable and incurable group**	***F*^*a*^**	***F*^*b*^**
**Gender**						
Male	7.33 ± 4.11	8.11 ± 4.81	7.93 ± 4.82	8.07 ± 4.46	10.958 (3,304159)***	199.812 (3,304159)***
Female	6.93 ± 3.77	7.86 ± 4.26	7.76 ± 4.33	8.74 ± 5.00		
**Grade**						
Fresher	7.07 ± 3.91	7.67 ± 4.27	7.56 ± 4.41	8.23 ± 5.38	5.498 (9,304151)***	206.412 (3,304151)***
Sophomore	7.13 ± 3.92	8.05 ± 4.62	7.95 ± 4.51	8.29 ± 5.10		
Junior	7.08 ± 3.92	8.11 ± 4.59	7.88 ± 4.57	8.41 ± 5.36		
Senior (4th and 5th)	7.02 ± 3.88	8.48 ± 4.71	8.31 ± 4.79	9.01 ± 5.26		
**Severe physical illness**						
Yes	7.66 ± 4.41	10.28 ± 5.17	8.48 ± 5.14	9.30 ± 7.12	8.642 (3,304159)***	17.047 (1,304159)***
No	7.09 ± 3.91	7.96 ± 4.50	7.82 ± 4.52	8.35 ± 5.24		
**History of mental illness**						
Yes	7.08 ± 4.39	8.10 ± 5.14	8.09 ± 5.01	11.71 ± 7.25	1.641 (3,304159)	11.639 (3,304159)***
No	7.09 ± 3.91	7.96 ± 4.50	7.82 ± 4.51	8.28 ± 5.19		
**Infection of friends**						
① Diagnosed or suspected infection	8.37 ± 4.29	9.63 ± 6.35	9.52 ± 5.53	9.42 ± 6.25	0.393 (6,304155)	31.361 (3,304155)***
② No one is infected	6.97 ± 3.87	7.78 ± 4.37	7.63 ± 4.41	8.11 ± 4.98		
③ Not sure	7.30 ± 3.98	8.17 ± 4.59	8.00 ± 4.60	8.46 ± 5.38		
**Infection of relative (non co-resident)**						
① Diagnosed or suspected infection	7.87 ± 4.34	8.57 ± 5.46	8.32 ± 5.41	9.22 ± 5.07	0.067 (6,304155)	11.347 (3,304155)***
② No one is infected	7.04 ± 3.88	7.87 ± 4.40	7.72 ± 4.38	8.15 ± 4.99		
③ Not sure	7.62 ± 4.23	8.50 ± 4.99	8.32 ± 5.06	8.76 ± 5.81		
**Infection of family (co-resident)**						
① Diagnosed or suspected infection	7.67 ± 4.23	8.86 ± 4.87	5.07 ± 4.88	9.67 ± 5.30	3.278 (6,304155)**	6.375 (3,304155)***
② No one is infected	7.06 ± 3.89	7.92 ± 4.46	7.75 ± 4.12	8.27 ± 5.09		
③ Not sure	8.06 ± 4.62	8.57 ± 5.17	8.68 ± 5.45	8.62 ± 5.91		
**Taking precautions**						
Yes	7.09 ± 3.91	7.94 ± 4.46	7.87 ± 4.45	8.44 ± 4.92	2.977 (3,304159)*	91.003 (3,304159)***
No	7.14 ± 4.16	8.30 ± 5.11	7.62 ± 4.83	8.24 ± 5.78		
**Family location**						
Rural areas	7.24 ± 0.01	8.05 ± 0.08	7.86 ± 0.08	8.18 ± 0.17	3.350 (6,304155)**	218.975 (3,304155)***
County town	7.03 ± 0.01	7.85 ± 0.10	7.74 ± 0.09	8.30 ± 0.18		
City	6.94 ± 0.01	7.98 ± 0.10	7.86 ± 0.08	8.63 ± 0.18		
**Family member (co-resident)**						
1–2 people	6.96 ± 0.04	7.491 ± 0.24	7.91 ± 0.22	8.35 ± 0.43	1.297 (9,304151)	116.433 (3,304151)***
3 people	6.93 ± 0.02	8.02 ± 0.13	7.86 ± 0.11	8.26 ± 0.23		
4 people	7.05 ± 0.01	7.93 ± 0.09	7.73 ± 0.08	8.38 ± 0.18		
5 people and above	7.20 ± 0.01	8.02 ± 0.08	7.87 ± 0.07	8.40 ± 0.16		
**Daily time spend on reading information about COVID-19**						
≤1 h	6.63 ± 0.01	7.45 ± 0.07	7.22 ± 0.06	7.69 ± 0.13	9.789 (6,304155)***	217.875 (3,304155)***
1–3 h	7.48 ± 0.01	8.50 ± 0.09	8.40 ± 0.08	8.71 ± 0.21		
≥3 h	7.93 ± 0.02	9.00 ± 0.16	9.24 ± 0.14	10.52 ± 0.26		

*p < 0.05; **p < 0.01; ***p < 0.001. ^a^Interaction test. ^b^Main effect text.

The perceptions^∗^gender interaction was significant, *F*(3,304159) = 10.958, *p* < 0.001. The simple main effect showed that the IES-6 scores of ‘unpreventable and incurable’ group was higher in female than in male (*p* < 0.01), while the IES-6 scores of ‘preventable and curable’ group was higher in male than in female (*p* < 0.001). The perceptions^∗^grade interaction was significant, *F*(9,304151) = 5.498, *p* < 0.001. In ‘preventable and incurable’ group and ‘unpreventable and curable’ group the difference between the fresher (*M* = 7.67, *SD* = 4.27; *M* = 7.56, *SD* = 4.41) and other grades was significantly greater. The perceptions^∗^history of mental illness interaction was significant, *F*(3,304159) = 8.642, *p* < 0.001. The simple main effect showed that the IES-6 scores of ‘unpreventable an incurable’ group was significantly higher in participants with a history of mental illness than participants without a history (*p* < 0.001). The perceptions^∗^infection of family interaction was significant, *F*(6,304155) = 3.278, *p* < 0.01. The simple main effect showed that the IES-6 scores of ‘preventable and curable’ group was significantly lower in participants whose families were diagnosed or suspected as being infected than in participants whose families were uninfected (*p* < 0.01) and those who did not know their infection status (*p* < 0.05).

The perceptions^∗^precautions interaction was significant, *F*(3,304159) = 2.977, *p* < 0.05. As hypothesized, the simple main effect showed that the IES-6 scores of ‘unpreventable and curable’ group was significantly higher in participants who had taken precautions than in participants without taking precautions (*p* < 0.05). The perceptions^∗^location interaction was significant, *F*(6,304155) = 3.350, *p* < 0.01. The simple main effect showed that the IES-6 scores of ‘preventable and curable’ group was significantly higher in rural than in city (*p* < 0.001). The perceptions^∗^reading time interaction was significant, *F*(6,304155) = 9.789, *p* < 0.01. In the group that spent 3 h or more on reading information about COVID-19 every day, the difference of psychological stress between the ‘preventable and curable’ group (*M* = 7.93, *SD* = 0.02) and ‘unpreventable and incurable’ group (*M* = 10.52, *SD* = 0.26) was even greater (*p* < 0.001).

## Discussion

Having spread to countries outside of China, COVID-19 has become a global threat. The rising numbers of cases and deaths, coupled with widespread quarantine measures, have the potential to create and spread fear, panic and distress among the general public. Guangdong was the second worst-affected province in China with more than 1,600 confirmed cases. After the Ministry of Education issued a notice on the postponement of the spring semester in Guangdong, it was thought that students might face a psychological challenge. Plans for interventions during public health emergencies such as the COVID-19 epidemic need to be based on an understanding of the factors related to the occurrence of mental health problems in this specific group.

The results of our cross-sectional study showed that 155,077 (50.9%) of the students surveyed reported stress symptoms, 1,565 (0.5%) reported poor mental health, and 9,752 (3.2%) reported poor sleep quality, which indicates that a substantial proportion of the students were distressed. Although quarantined students experienced PTSS symptoms, the scales that were used to measure these symptoms were not sufficient to confirm a diagnosis of PTSD. To confirm such a diagnosis, structured diagnostic interviews would have been required. As the survey was anonymous, this was not possible. An average score of ≥1.09 on the IES-6 scale was used to estimate the prevalence of PTSS in our study. While other cutoff points might have been used to estimate PTSD, the important findings of this study are the risk factors that were identified as likely to increase PTSS, rather than the absolute prevalence of PTSD.

In the face of a large-scale epidemic, media broadcasts are one of the most effective methods of reducing epidemic-induced panic. On the one hand, media broadcasts have provided people with sufficient information for a good understanding of the prevalence of COVID-19 and its nature. In China, official departments strove to improve the public’s awareness of prevention and intervention strategies by providing daily updates about surveillance and active cases on websites and social media (Bao et al., 2020). On the other hand, many self-publicists and netizens also release and recirculate related information which might include misinformation and rumors on social media, such as WeChat and Weibo, which can also lead to (mis)information overload (Bontcheva et al., 2013) and subsequently give rise to mental health problems. In addition, because people paid attention to the information that was updated daily with new cases and fatalities, the severity of COVID-19 was reinforced and the level of public panic was heightened. This might explain the varying risk perceptions of COVID-19 in our results, and also explain that the psychological stress significantly increased when participants read relevant information for 3 h or more per day.

This study showed that reading COVID-19 relevant information for 3 h or more every day was more likely to have negative perception. Similar to our results, a study focused on the MERS outbreak in South Korea showed that social media exposure might have been positively related to the formation of risk perceptions during the outbreak (Choi et al., 2017). Leppin and Aro (2009) suggested that in the first stage of the SARS outbreak risk perception was strongly influenced by the nature of the hazard and the way that the media reported on the cases and fatality incidents. The current study of more than 300,000 students found that 290,148 (95.4%) of the sample considered COVID-19 as preventable and curable, 6,979 (2.2%) considered it as unpreventable but curable, 5,549 (1.8%) considered it as preventable but incurable, and only 1,491 (0.5%) considered it as unpreventable and incurable. Overall, it seems that the students tended to have a more positive mindset after the occurrence of the COVID-19 outbreak; however, around 50% of the students showed stress symptoms. It is worth noting that students in the ‘unpreventable and incurable’ group were the least likely to take precautions. In terms of psychological processes, one explanation for the diversity of responses is that in some populations a very high risk perception, rather than mobilizing people to take action, might evoke a sense of helplessness which could paralyze rather than stimulate protective behaviors. For threats like a pandemic, the effectiveness of countermeasures must remain unclear in the initial absence of a vaccine, such that no-action responses might be particularly prone to occur (Leppin and Aro, 2009).

A significant association was noted between the occurrences of stress symptoms and perceptions of COVID-19. The ‘unpreventable and incurable’ group had the higher stress levels and a higher prevalence of the symptoms that may indicate a PTSD diagnosis. Additionally, a significant connection was found between self-perceived mental health and perceptions of COVID-19, and also between sleep quality and the perceptions. The ‘unpreventable and incurable’ group had the poorest mental health levels and the poorest sleep quality. Research has found that a higher perceived risk of being infected by coronavirus and a higher perceived level of harm led to significantly higher anxiety levels among the general public in China (Qian et al., 2020), suggesting that risk perceptions of COVID-19 can affect the mental health of a general population. In the current study, negative perceptions could have made the epidemic seem more catastrophic and high-risk, which in turn made students feel helpless and led them to underestimate their ability to cope. Such consequences are more likely to cause psychological stress. Other research on the stress caused by epidemic diseases has revealed that the perception of SARS played a mediatory role between stress caused by external sources and emotional and behavioral responses (Qian et al., 2005). Moreover, the current study also found that the ‘unpreventable and incurable’ group had a higher possibility of experiencing epidemic-related stress events (such as infection or suspected infection with COVID-19 in friends, relatives and families), as well as a higher possibility of susceptible factors (such as severe physical illness, poor physical health, a history of mental illness or a history of counseling), which means that all those who had encountered stress events or exhibited susceptible factors had higher stress levels. Hence, since the ‘unpreventable and incurable’ group also suffered from their physical illness, mental impact, or epidemic events, they were more likely to experience stress symptoms. In addition, a tendency toward an optimistic bias should influence perceptions so that personal ratings are generally lower than those for the overall population (Leppin and Aro, 2009), which could be another explanation for the ‘preventable and curable’ group having lower psychological stress levels.

Further analysis revealed that there was an interaction between demographic characteristics and perceptions of COVID-19. The demographic characteristics influenced the relationship between perception types and levels of stress symptoms. More females than males perceived COVID-19 to be ‘unpreventable and curable,’ whereas more males than females perceived COVID-19 to be ‘unpreventable and incurable.’ There was an interaction between gender and perception. We found that females with an ‘unpreventable and incurable’ perception suffered more of a psychological impact than men with the same perception, suggesting that females are much more vulnerable to stress. These results are in accordance with previous research which found that the level of stress and psychological distress is higher in female than in male college students (Backović et al., 2012; Saleh et al., 2017; Gao W. et al., 2020). During the COVID-19 epidemic, the emotional response index for females was found to be higher than that for males (Qiu et al., 2020). Other research found that females were more easily worried than males about being infected during an epidemic and were more fearful that the epidemic was hard to control (Furer et al., 1997), suggesting that if females think that COVID-19 infection can’t be prevented, the worry will be more obvious, which may explain why the females with an ‘unpreventable and incurable’ perception suffered more of a psychological impact. On the other hand, some studies of stress among students found that male students reported higher stress levels than females (Shashidhar, 2003; Ahern and Norris, 2011). In the current study, we found that the males with the ‘preventable and curable’ perception suffered more of a psychological impact than the females with the same perception. This may be because females are more likely than males to expect and accept the help of others; when disasters occur, females are more likely to be positively affected by the social support systems that might provide them with relief from psychological stress (Liu et al., 2004).

Young people tend to obtain a large amount of information from social media which can easily trigger stress if too much time is spent on information about the corona epidemic. Perceived risk of infection and perceived severity of the disease as well as information reliability were found to be important factors associated with the psychological and behavioral responses of people in China (Qiu et al., 2020). Research on psychological stress among college students of different ages has indicated a positive correlation between psychological stress and age (Fornes-Vives et al., 2012; Voltmer et al., 2012). Our results were similar to these research in finding that younger students and freshers were relatively more optimistic about their perception of the disease and had lower stress levels during the COVID-19 epidemic. On the one hand, the consideration that the highest mortality rate was occurring among the elderly might have led younger people to have more confidence in their autoimmunity and to have better psychological tolerance for such emergencies. On the other hand, higher-grade students might have entered a reflective and confused period, and have a higher self-awareness of their health and future. Due to the impact of the epidemic, senior students in particular will face a series of problems and stresses, such as difficulties in progressing a graduation thesis, delays in graduation and anxieties about employment. It was also found that there was a higher risk of depression during COVID-19 among those aged 21–40 years compared to those who were under 20 (Gao J. et al., 2020).

Participants with a severe physical had a more negative perception of COVID-19 and were more vulnerable to psychological stress in the two preventable groups. This may be related to a higher mortality rate for people with a previous medical history and lower immunity. Judgments on the personal likelihood of contracting an infectious disease are subject to considerations of individual immune competence and host resistance. There was no difference between the two unpreventable groups in the levels of psychological stress, which were related to students’ perceptions of the virus and their demographic characteristics. Students who believe that COVID-19 infection is not preventable, regardless of physical illness, may feel that they have the same probability of contracting pneumonia. The manifestation of this panic mood may be related to the body’s normal protective response to the stress caused by an epidemic (Maunder et al., 2003). A cognitive appraisal framework assumes that it is mostly perceptions that give rise to emotions (Roseman, 1996). In the case of a long-term chronic disease, emotions such as fear are likely to be less imminent and therefore secondary to more rational reflections about gains and losses related to protective behavior. However, in an acute threat situation such as an influenza pandemic, emotional aspects might gain a more immediate importance. This is all the more likely if, during the early stages of an outbreak, experts are unable to make more than tentative statements and provide partly contradictory prognoses and recommendations. Unlike the individual long-term development of a chronic disease, where there is an extended timeframe for changes in behavior, an outbreak situation creates a massive, collective and acute threat, and people tend to feel more out of control (Leppin and Aro, 2009). Besides, this study revealed that there was an interaction between mental illness history and perceptions of COVID-19, participants with mental illness were significantly more vulnerable to psychological stress in ‘unpreventable and incurable’ group. This may be related to the lower psychological resistance and higher susceptibility of participants with a history of mental illness. Negative perception will further aggravate low psychological resistance and high susceptibility, which would have led to worries about being infected with COVID-19.

The interaction between perceptions and family location was significant. In ‘preventable and curable’ group, students living in city had lower psychological stress than students living in rural during COVID-19. Likewise, a study has found that living in urban areas, in contrast to rural areas, was protective factor against anxiety of college students (Cao et al., 2020). This might be explained by the imbalance of economic, cultural, and educational resources between urban and rural areas. The urban economy is relatively prosperous and provides citizens with better material security. Similarly, the sanitary conditions in cities are better than in towns and villages, which decreases the chances of surviving the virus. Cities also have excellent educational resources, and they have made great efforts to publicize knowledge on how to prevent the epidemic, which attracts attention to the measures taken to stop the epidemic.

PTSD is an anxiety disorder that is characterized by avoiding stimuli associated with traumatic events, by re-experiencing the trauma (intrusion), and by hyperarousal. This disorder may develop after exposure to traumatic events that involve a life-threatening component, and if the trauma is perceived to be a personal assault the vulnerability of the person to developing PTSD may increase (Breslau et al., 1998; Pietrzak et al., 2014). The presence of more stress symptoms in students acquainted with friends, relatives or families who were confirmed or suspected as being infected with COVID-19 may indicate a greater perceived self-risk, compared to students who did not have this personal connection. In contrast, students in the ‘unpreventable and curable’ group whose families were diagnosed or suspected of infection suffered less stress than students who had no family infection. The principle of consistency states that when two or more simultaneously active cognitive structures are logically inconsistent, arousal is increased, which activates processes with the expected consequence of increasing consistency and decreasing arousal (Kampen, 2019). Brown (2005) pointed to belief modification as a means of reducing cognitive dissonance, which might cancel out any protective motivational impulses of risk appraisal. We speculate that students who perceived COVID-19 infection to be unpreventable would be more fearful of becoming infected. When faced with infection in the family, which increased the degree of perceived self-risk and panic, students modified their cognition in order to reduce emotional arousal. Students rationalized and accepted the fact that members of their family were infected by persuading themselves that COVID-19 is not preventable, and they had confidence that their families could be cured. After this cognitive disruption, there was less psychological stress in the attempt to reduce dissonance.

In the two preventable groups, the level of psychological stress was slightly lower among students who took precautions than those who did not, although the results were not significant. In contrast, as hypothesized, in the two unpreventable groups, the level of stress was higher in the students who took precautions, and there was a significant difference in the ‘unpreventable and curable’ group. The theory of cognitive dissonance often involves an inconsistency between an individual’s existing attitude and their behavior in a certain social context, which has been described as an aversive motivational state (Brown, 2005). Students who believed that COVID-19 infection was unpreventable took precautions because of a high level of anxiety, and this inconsistency between cognition and behavior (i.e., cognitive dissonance) led to higher levels of psychological stress. In order to alleviate this pressure according to the principle of consistency, adjusting students’ perception of unpreventable COVID-19 would be an effective method.

A web-based survey was conducted to assess the psychological stress levels among students during the COVID-19 outbreak in Guangdong. This approach has several advantages—in particular, high efficiency and low cost. The major limitations of the survey are, firstly, the lack of information about the non-respondents. There may be selection bias, and as all the data were self-reported the results may reflect a social desirability bias. However, systematic differences between respondents and non-respondents cannot be ascertained accurately. This might limit the interpretation of the results, but the large sample size can compensate for this. Secondly, our study was a cross-sectional design and it is difficult to make causal inferences. People were likely to experience four emotional phases or responses during the outbreak: shock/disbelief; a strong emotional response; acceptance; and recovery (Kowalskia and Kalayjian, 2001). Our study was conducted during the first stage of the outbreak, when students’ perceptions might have been strongly influenced. Future research may continue to detect changes in students’ perceptions at different stages of the epidemic. Thirdly, we assessed only the psychological stress levels of college students in Guangdong province after the COVID-19 outbreak (the worst-affected province after Hubei), which limits the scope of the conclusions.

Despite these limitations, the results of this survey allow for the generation of hypotheses that require further exploration. The results demonstrate the psychological impact on college students when faced with a severe infectious disease, and show that negative perceptions can result in considerable psychological distress in the form of PTSD symptoms. A review of longitudinal studies found that during the initial phases of the SARS outbreak in 2003 risk perception showed a steady increase and only stabilized in later phases (Qian et al., 2020), which suggests that the psychological impact on students will continue for a period of time. Public health officials, infectious disease physicians, psychiatrists and psychologists need to be made aware of this issue. They must be prepared to provide additional support to students who are at increased risk of the adverse psychological effects when the COVID-19 broke out and students were quarantined instead of attending school.

Our findings also yield several important public implications. Firstly, providing the students with reliable, accurate and acceptable information is crucial for addressing the psychological effects of contagious disease outbreaks. Students’ negative perceptions of whether the disease could be treated and infection prevented might have led to significantly sustained psychological stress. Media companies should manage scientifically the information that they release, focus on learning more about the psychological changes in the general public, and determine whether the public can accept the information when publishing. Additionally, media companies and related organizations need to combat “infodemics” using a variety of strategies, including monitoring and filtering out misinformation, clarifying rumors and conducting live Q&A interviews with experts. Secondly, colleges and universities should launch psychological counseling services in a timely manner and establish a system of online psychological assistance to enable students with severe psychological stress to adjust their mental state in a timely manner and to maintain a basic psychological balance. This study provides a basis for understanding the characteristics of students suffering psychological stress as a result of a public health emergency. Colleges and universities could provide targeted psychological services for different characteristics of the student population. At the same time, it is important to note that the focus of the assistance should be different during different stages of an epidemic. In the early stage, the assistance should focus on psychological stress. As the epidemic develops, the service should focus on psychological and behavioral disorders, alerting students to the potential disorders caused by the epidemic, such as phobias, anxiety and depression. Students with an obvious psychological impact in the early stage may be the focus for later evaluation, psychological assistance, and a short-term and long-term follow-up. Lastly, in the future, health education courses for common disasters could be established in colleges and universities to enhance the normal stress resistance of college students against disasters and encourage them to be a backup force for health education in the event of future disasters.

## Conclusion

To conclude, college students’ perceptions of COVID-19 were correlated with psychological stress, self-perceived mental health and sleep quality. And the degree of stress also varied with types of perception. Secondly, as hypothesized, the study found that when students’ behavior was inconsistent with perception, the psychological impact was higher than students whose behaviors were consistent with perception. Furthermore, as this is the large sample study on the immediate perceptions of COVID-19 among college students, understanding these factors will help us to design intervention programs with the aim of alleviating stress among students.

## Data Availability Statement

The datasets presented in this article are not readily available because the dataset involves 85 colleges and universities, and sharing requires the approval of all schools. Requests to access the datasets should be directed to JZ, mingtian@smu.edu.cn.

## Ethics Statement

The studies involving human participants were reviewed and approved by the institutional ethics board of Southern Medical University. Written informed consent from the participants’ legal guardian/next of kin was not required to participate in this study in accordance with the national legislation and the institutional requirements.

## Author Contributions

JZ designed the study. XL, RC, JC, SL, and ST participated in the data collection and analysis, and writing and revising the manuscript. SL and LL participated in the writing and revising of the manuscript. All authors contributed to the article and approved the submitted version.

## Conflict of Interest

The authors declare that the research was conducted in the absence of any commercial or financial relationships that could be construed as a potential conflict of interest.

## Footnotes

1 https://www.who.int/news-room/detail/30-01-2020-statement-on-the-second-meeting-of-the-international-health-regulations-(2005)-emergency-committee-regarding-the-outbreak-of-novel-coronavirus-(2019-ncov)

2 https://news.qq.com/zt2020/page/feiyan.htm#/global

3 http://www.nhc.gov.cn/xcs/zhengcwj/202001/6adc08b966594253b2b791be5c3b9 467.shtml
